# Supplementary material for: Nutrient-Derived Beneficial for Blood Pressure Dietary Pattern Associated with Hypertension Prevention and Control: Based on China Nutrition and Health Surveillance 2015–2017
Source: Nutrients. 2022 Jul 28;14(15):3108. doi: 10.3390/nu14153108 (PMC9370233; doi:10.3390/nu14153108)
Supplement: Supplementary file 1 [file nutrients-14-03108-s001.zip › Supplemental_finalproof1.pdf]

Table S1. Characteristics of dietary patterns derived from RRR

Dietary pattern	DP1	DP2	DP3	DP4	DP5	DP6	DP7
Explained variation of response variables (%)	66.08	8.172	5.819	4.33	2.638	0.5	0.389
Explained variation of food items (%)	5.725	3.879	4.35	2.21	3.686	3.39	3.441
**Factor loading**							
Refined grains	-0.314	0.177	0.187	0.366		-0.167	
Coarse cereals	0.136	0.281	-0.364	0.23	-0.242	0.15	
Legumes and related products	0.145	0.148	-0.129	0.133	0.15	-0.114	-0.276
Nuts and seeds	0.158	0.14	-0.342		-0.276	0.354	0.151
Tubers		0.145		0.112	-0.105		-0.24
Fried staples			-0.106				
Fresh vegetables	0.537	0.182	0.44	-0.115		-0.128	
Dried vegetables						-0.247	0.231
Fermented vegetables					-0.157	-0.11	-0.231
Mushrooms/edible fungi	0.344		-0.445		0.64		
Seaweeds	0.196		-0.19		0.254		0.169
Fresh fruits	0.37	0.164	0.127	-0.473	0.175	0.305	
Dried fruits	0.173		-0.254			-0.166	0.407
Dairy products	0.31	-0.766		-0.117	-0.222		0.105
Pork		-0.143	0.34	0.597	0.402		0.108
Beef, lamb, and other meats		-0.157			0.1	0.375	-0.317
Poultries		-0.141		0.116	0.159	0.253	-0.166
Processed meats							
Organs				0.187	0.111	0.205	
Aquatic products	0.142	-0.172		0.224		-0.13	-0.371
Fresh eggs	0.189	-0.185		0.101		0.488	
Processed eggs						0.106	
Western staples	0.109	-0.112					
Snacks							
100% vegetable and fruit juice				-0.112			-0.232
Coffee						-0.181	-0.39
Sweetened beverages							
Alcohol	-0.106					-0.132	0.118
**Correlations with response nutrients**							
Thiamin	0.549*	0.168 *	0.353*	0.515*	0.042*	-0.002	0.097*
Riboflavin	0.863*	-0.193*	0.046*	0.1*	0.084*	0.191*	0.019*
Vitamin C	0.806*	0.285*	0.414*	-0.231*	-0.018*	0.061*	-0.008
Calcium	0.916*	-0.038*	0.038*	-0.085*	-0.235*	-0.009*	-0.029*
Magnesium	0.830*	0.402*	-0.201*	0.116*	-0.365*	0.129*	-0.064*
Potassium	0.928*	0.093*	-0.011*	0.035*	-0.006	0.047*	-0.109*
Dietary fiber	0.889*	0.409*	-0.136*	-0.065*	-0.104*	0.101*	0.026*

* Indicates *p* value <0.05.

Table S2. Daily food intakes of participants by quintiles of BBP-diet score

Food item	Quintile				
	Q1	Q2	Q3	Q4	Q5
Refined grains*	304.93 (202.47, 456.99)	303.29 (207.12, 450)	300 (200, 400)	242.74 (156.99, 328.49)	170.96 (112.74, 250)
Coarse grains*	0 (0, 3.95)	0 (0, 9.86)	1.64 (0, 18.08)	4.27 (0, 27.85)	9.86 (0, 34.19)
Legumes and related products*	14.36 (4.03, 40.34)	21.49 (7.59, 53.41)	28.49 (10.39, 65.05)	37.07 (14.66, 80.74)	44.21 (18.99, 91.35)
Nuts and seeds*	0 (0, 2.63)	0.55 (0, 6.58)	1.1 (0, 8.55)	1.64 (0, 14.25)	2.85 (0, 14.25)
Tubers*	7.12 (0.41, 28.49)	13.15 (1.64, 42.74)	14.25 (3.29, 50)	21.37 (4.93, 56.99)	25.64 (7.12, 60)
Fried staples*	0 (0, 3.45)	0 (0, 6.58)	0 (0, 7.56)	0.41 (0, 8.55)	0 (0, 6.9)
Fresh vegetables*	100 (50, 200)	200 (100, 300)	284.93 (160, 400)	300 (200, 500)	400 (250, 600)
Dried vegetables*	0 (0, 0)	0 (0, 0.05)	0 (0, 0.11)	0 (0, 0.14)	0 (0, 0.16)
Fermented vegetables*	0.66 (0, 7.89)	0.82 (0, 8.55)	0.82 (0, 8.55)	0.66 (0, 8.55)	0 (0, 7.12)
Mushrooms/edible fungi*	2.05 (0, 8.36)	5.63 (0.68, 16.71)	9.37 (1.64, 25.64)	14.25 (3.29, 38.11)	28.49 (7.4, 74.79)
Seaweeds*	0 (0, 1.32)	0.27 (0, 2.83)	0.64 (0, 3.95)	1.26 (0, 6.41)	2.14 (0, 9.21)
Fresh fruits*	19.73 (3.95, 50)	42.74 (14.25, 100)	60 (21.37, 142.47)	100 (29.92, 199.45)	150 (56.99, 250)
Dried fruits*	0 (0, 0)	0 (0, 0.11)	0 (0, 0.41)	0 (0, 1.15)	0 (0, 3.29)
Dairy products*	0 (0, 0)	0 (0, 7.47)	0 (0, 28.49)	0 (0, 75.72)	38.85 (0, 200)
Pork*	28.49 (7.12, 71.23)	39.89 (13.15, 100)	34.19 (12.82, 85.48)	28.49 (11.4, 64.11)	21.37 (7.12, 50)
Beef, lamb, and other meats*	0 (0, 3.23)	0.55 (0, 4.79)	0.82 (0, 6.58)	1.64 (0, 9.32)	3.29 (0, 14.25)
Poultries*	1.48 (0, 6.58)	3.29 (0, 9.86)	3.29 (0, 9.86)	3.29 (0, 12.82)	3.29 (0, 13.15)
Processed meats*	0 (0, 0)	0 (0, 0.16)	0 (0, 0.41)	0 (0, 0.82)	0 (0, 0.66)
Organs*	0 (0, 0.16)	0 (0, 0.55)	0 (0, 0.66)	0 (0, 0.66)	0 (0, 0.22)
Aquatic products*	2.36 (0, 10.68)	6.58 (0.41, 19.73)	7.95 (0.82, 28.49)	9.86 (1.37, 31.34)	14.25 (2.74, 41.95)
Fresh eggs*	10.68 (2.3, 25.64)	15.67 (6.58, 34.19)	21.37 (7.84, 50)	25.64 (9.86, 50)	31.34 (14.25, 55)
Processed eggs*	0 (0, 0.16)	0 (0, 0.68)	0 (0, 1.1)	0 (0, 1.37)	0 (0, 1.32)
Western staples*	0 (0, 0.68)	0 (0, 3.29)	0 (0, 5.26)	0 (0, 7.12)	0.21 (0, 12.82)
Snacks*	0 (0, 0.33)	0 (0, 1.1)	0 (0, 1.64)	0 (0, 2.1)	0 (0, 2.14)
100% vegetable and fruit juice*	0 (0, 0)	0 (0, 0)	0 (0, 0)	0 (0, 0)	0 (0, 0)
Coffee*	0 (0, 0)	0 (0, 0)	0 (0, 0)	0 (0, 0)	0 (0, 0)
Sweetened beverages*	0 (0, 3.42)	0 (0, 7.12)	0 (0, 8.22)	0 (0, 8.49)	0 (0, 4.93)
Alcohol*	0 (0, 3.82)	0 (0, 2.86)	0 (0, 2.19)	0 (0, 1.32)	0 (0, 0.66)

* Indicates p value <0.001.

Table S3. Daily nutrient intakes of participants by quintiles of BBP-diet score

Nutrient	Quintile				
	**Q1**	**Q2**	**Q3**	**Q4**	**Q5**
Energy (Kcal)*	2270.86 (1692.71, 2989.74)	2291.58 (1772.85, 2960.32)	2218.68 (1727.32, 2851.82)	2135.9 (1665.87, 2738.16)	1991.46 (1547.26, 2572.73)
Protein (g)*	52.82 (37.21, 74.31)	62.15 (45.94, 84.53)	65.61 (48.02, 88.05)	67.55 (50.06, 90.9)	70.46 (52.68, 94.13)
Fat (g)*	83.72 (59.56, 113.24)	82.42 (60.09, 109.89)	79.8 (58.92, 107.29)	77.74 (57.53, 103.93)	71.46 (52.85, 95.63)
Carbohydrate (g)*	279.13 (196.18, 402.28)	296.85 (219.55, 410.6)	293.09 (215.64, 401.92)	287.45 (209.9, 379.01)	268.28 (199.56, 355.74)
Dietary fiber (g)*	6.16 (4.28, 8.36)	8.91 (6.73, 11.77)	10.92 (8.19, 14.54)	13.36 (9.94, 17.86)	17.72 (12.89, 24.68)
Vitamin A (μgRAE)*	138.37 (87.23, 203.32)	222.35 (161.93, 303.75)	281.18 (206.07, 375.42)	341.89 (254.02, 449.01)	449.89 (335.33, 609.34)
Thiamine (mg)*	0.91 (0.64, 1.3)	1.06 (0.78, 1.46)	1.1 (0.82, 1.49)	1.12 (0.84, 1.5)	1.16 (0.87, 1.53)
Riboflavin (mg)*	0.48 (0.34, 0.67)	0.65 (0.49, 0.87)	0.76 (0.57, 1.01)	0.88 (0.66, 1.16)	1.12 (0.83, 1.52)
Niacin (mg)*	10.76 (7.44, 15.52)	12.62 (9.12, 17.55)	12.96 (9.36, 17.78)	13.1 (9.58, 17.74)	13.46 (9.92, 18.31)
Vitamin C (mg)*	34.29 (20.83, 50.91)	62.32 (43.74, 83.92)	80.6 (58.28, 106.78)	100.81 (71.98, 134.02)	137.91 (99.18, 193.67)
Vitamin E (mg)*	26.54 (17.83, 38.58)	27.19 (19.23, 37.36)	28.6 (20.42, 38.55)	29.87 (21.58, 40.27)	30.99 (22.75, 41.78)
Calcium (mg)*	216.12 (151.98, 297.55)	323.44 (244.76, 424.53)	401.81 (305.97, 529.4)	500.69 (377.03, 655.21)	680.91 (505, 921.93)
Potassium (mg)*	1212.91 (875.15, 1653.86)	1635.62 (1255.44, 2154.94)	1906.41 (1461.62, 2504.08)	2218.51 (1703.36, 2913.02)	2865.35 (2143.53, 3811.11)
Sodium (mg)*	3792.42 (2685.74, 5494.97)	3573.24 (2577.34, 4968.85)	3471.28 (2550.44, 4806.29)	3397.66 (2526.66, 4668.47)	3242.09 (2394.57, 4391.57)
Magnesium (mg)*	228.89 (165.3, 311.44)	283.82 (215.42, 374.59)	315.51 (238.74, 417.29)	348.8 (263.83, 464.16)	400.89 (302.99, 534.97)
Iron (mg)*	11.84 (8.65, 15.74)	15.07 (11.62, 19.67)	17.24 (13.22, 22.6)	19.7 (15.06, 25.83)	24.51 (18.15, 33.07)
Zinc (mg)*	7.57 (5.31, 10.62)	8.84 (6.55, 12.04)	9.25 (6.84, 12.36)	9.66 (7.21, 12.75)	10.39 (7.83, 14)
Selenium (μg)*	28.1 (19.32, 40.61)	34.44 (24.62, 47.89)	37.44 (26.52, 52.03)	39.57 (28.12, 54.44)	42.09 (30.1, 57.9)

* Indicates p value <0.001

Table S4. Subgroup analysis of associations between BBP-diet score and risk of hypertension

Subgroup	N (%)	Quintile (OR, 95% CI)*, †					*p* for interaction
		Q1	Q2	Q3	Q4	Q5	
Gender							0.7001
Male	28,388 (45.97%)	reference	0.935 (0.865, 1.01)	0.913 (0.843, 0.989)	0.83 (0.763, 0.902)	0.819 (0.747, 0.898)	
Female	33,359 (54.03%)	reference	1.008 (0.925, 1.098)	0.964 (0.884, 1.05)	0.961 (0.882, 1.047)	0.906 (0.831, 0.989)	
Age							0.1254
18~ < 30	5503 (8.91%)	reference	1.473 (1.047, 2.072)	0.912 (0.635, 1.308)	1.066 (0.743, 1.529)	1.132 (0.769, 1.666)	
30~ < 45	13,537 (21.92%)	reference	0.976 (0.843, 1.131)	0.894 (0.77, 1.037)	0.877 (0.753, 1.021)	0.901 (0.768, 1.058)	
45~ < 60	23,176 (37.53%)	reference	0.957 (0.878, 1.044)	0.95 (0.87, 1.037)	0.891 (0.815, 0.974)	0.881 (0.8, 0.969)	
≥ 60	19,531 (31.63%)	reference	0.939 (0.854, 1.032)	0.954 (0.866, 1.05)	0.893 (0.809, 0.986)	0.784 (0.709, 0.867)	
Physical activity							0.5472
Low	14,590 (23.63%)	reference	0.999 (0.891, 1.119)	0.971 (0.864, 1.091)	0.954 (0.846, 1.076)	0.834 (0.733, 0.949)	
Medium	15,320 (24.81%)	reference	0.946 (0.832, 1.076)	0.955 (0.842, 1.083)	0.842 (0.741, 0.956)	0.795 (0.699, 0.904)	
High	31,837 (51.56%)	reference	0.958 (0.885, 1.036)	0.908 (0.838, 0.983)	0.873 (0.804, 0.948)	0.861 (0.79, 0.94)	
Current smoker							0.1202
No	45,746 (74.09%)	reference	0.996 (0.928, 1.069)	0.951 (0.886, 1.021)	0.923 (0.859, 0.991)	0.852 (0.792, 0.917)	
Yes	16,001 (25.91%)	reference	0.915 (0.828, 1.011)	0.903 (0.814, 1.003)	0.798 (0.714, 0.891)	0.863 (0.762, 0.978)	
Diabetes							<0.0001
No	56,478 (91.47%)	reference	0.978 (0.92, 1.038)	0.9 (0.894, 1.01)	0.905 (0.85, 0.963)	0.858 (0.803, 0.917)	
Yes	5269 (8.53%)	reference	0.844 (0.685, 1.04)	0.747 (0.609, 0.916)	0.696 (0.566, 0.856)	0.654 (0.532, 0.804)	
Hyperlipidemic							0.5423
No	38,221 (61.9%)	reference	0.975 (0.906, 1.05)	0.95 (0.881, 1.024)	0.889 (0.823, 0.96)	0.827 (0.762, 0.897)	
Yes	23,526 (38.1%)	reference	0.962 (0.876, 1.055)	0.918 (0.836, 1.007)	0.882 (0.802, 0.968)	0.859 (0.779, 0.947)	

* Abbreviations: OR, odds ratio; CI, confidence interval.

† OR for risk of hypertension is shown by comparing the highest quintile (Q5) with the lowest quintile (Q1) with the full-adjusted logistic model but without the variable used for dividing the subgroups.

Table S5. Sensitivity analysis for further adjustment or exclusion

Dietary pattern	Sensitive analysis	Quintile				
		Q1	Q2	Q3	Q4	Q5
BBP-diet	Main model*	reference	0.968 (0.913, 1.025)	0.935 (0.882, 0.992)	0.885 (0.834, 0.939)	0.842 (0.791, 0.896)
	Further adjusted for DM and hyperlipidemic	reference	0.969 (0.915, 1.027)	0.934 (0.881, 0.991)	0.885 (0.834, 0.94)	0.835 (0.785, 0.889)
	Excluded participants who didn't give household income	reference	0.963 (0.904, 1.027)	0.937 (0.879, 0.999)	0.894 (0.838, 0.955)	0.853 (0.797, 0.913)
	Excluded participants who were prediagnosed with HTN	reference	0.981 (0.921, 1.044)	0.941 (0.883, 1.003)	0.868 (0.813, 0.927)	0.824 (0.769, 0.884)
DASH diet	Main model*	reference	1.006 (0.949, 1.067)	0.964 (0.909, 1.022)	1.005 (0.949, 1.07)	0.912 (0.854, 0.973)
	Further adjusted for DM and hyperlipidemic	reference	1.003 (0.913, 1.063)	0.964 (0.909, 1.022)	1.004 (0.943, 1.069)	0.906 (0.846, 0.964)
	Excluded participants who didn't give household income	reference	1.007 (0.944, 1.074)	0.956 (0.897, 1.02)	1.014 (0.948, 1.086)	0.92 (0.857, 0.987)
	Excluded participants who were prediagnosed with HTN	reference	1.008 (0.946, 1.074)	0.945 (0.887, 1.007)	1.001 (0.935, 1.072)	0.88 (0.819, 0.946)

* Adjusted for age, gender, BMI, living area, education level, income, marital status, physical activity, sedentary behavior, sleep duration, current smoker (Yes/No), excessive drinking (Yes/No), second-hand smoke (Yes/No), medical examination within one year (Yes/No), family history of HTN (Yes/No), daily energy intake (Kcal/d), and daily sodium intake (mg/d).
